# Predictive Immunological, Virological, and Routine Laboratory Markers for Critical COVID-19 on Admission

**DOI:** 10.1155/2021/9965850

**Published:** 2021-08-02

**Authors:** Mercedes García-Gasalla, Juana M. Ferrer, Pablo A. Fraile-Ribot, Adrián Ferre-Beltrán, Adrián Rodríguez, Natalia Martínez-Pomar, Luisa Ramon-Clar, Amanda Iglesias, Inés Losada-López, Francisco Fanjul, Joan Albert Pou, Isabel Llompart-Alabern, Nuria Toledo, Jaime Pons, Antonio Oliver, Melchor Riera, Javier Murillas

**Affiliations:** ^1^Internal Medicine, Hospital Universitari Son Espases-Institut d'Investigació Sanitària Illes Balears (IdISBa), Palma, Balearic Islands, Spain; ^2^Immunology, Hospital Universitari Son Espases-Institut d'Investigació Sanitària Illes Balears (IdISBa), Palma, Balearic Islands, Spain; ^3^Microbiology, Hospital Universitari Son Espases-Institut d'Investigació Sanitària Illes Balears (IdISBa), Palma, Balearic Islands, Spain; ^4^Internal Medicine, Hospital Universitari Son Llàtzer-Institut d'Investigació Sanitària Illes Balears (IdISBa), Palma, Balearic Islands, Spain; ^5^Pneumology, Hospital Universitari Son Espases-Institut d'Investigació Sanitària Illes Balears (IdISBa), Palma, Balearic Islands, Spain; ^6^CIBER de Enfermedades Respiratorias, Hospital Universitari Son Espases-Institut d'Investigació Sanitària Illes Balears (IdISBa), Palma, Balearic Islands, Spain; ^7^CIBERES, Madrid, Spain; ^8^Análisis Clínicos, Hospital Universitari Son Espases, Palma, Balearic Islands, Spain

## Abstract

**Background:**

Early identification of COVID-19 patients at risk of critical illness is a challenging endeavor for clinicians. We aimed to establish immunological, virological, and routine laboratory markers, which, in combination with clinical information, may allow identifying such patients.

**Methods:**

Blood tests to measure neutrophil/lymphocyte ratio (NLR) and levels of ferritin, CRP, D-dimer, complement components (C3 and C4), cytokines, and lymphocyte subsets, as well as SARS-Cov-2 RT-PCR tests, were performed in COVID-19-confirmed cases within 48 hours of admission. RT-PCR cycle threshold (Ct) values from oropharyngeal or nasopharyngeal swabs were determined on the day of admission. Symptom severity was categorized as mild (grade 1), severe (grade 2), or critical (grade 3).

**Results:**

Of 120 patients who were included, 49 had mild, 32 severe, and 39 critical COVID-19. Levels of ferritin >370 ng/mL (OR 16.4, 95% CI 5.3–50.8), D-dimer >440 ng/mL (OR 5.45, 95% CI 2.36–12.61), CRP >7.65 mg/dL (OR 11.54, 95% CI 4.3–30.8), NLR >3.77 (OR 13.4, 95% CI 4.3–41.1), IL-6 >142.5 pg/mL (OR 8.76, 95% CI 3.56–21.54), IL-10 >10.8 pg/mL (OR 16.45, 95% CI 5.32–50.81), sIL-2r*α* (sCD25) >804.5 pg/mL (OR 14.06, 95% CI 4.56–43.28), IL-1Ra >88.4 pg/mL (OR 4.54, 95% CI 2.03–10.17), and IL-18 >144 pg/mL (OR 17.85, 95% CI 6.54–48.78) were associated with critical COVID-19 in the univariate age-adjusted analysis. This association was confirmed in the multivariate age-adjusted analysis only for ferritin, CRP, NLR, IL-10, sIL-2r*α*, and IL-18. T, B, and NK cells were significantly decreased in critical patients. SARS-CoV-2 was not detected in blood except in 3 patients who had indeterminate results. RT-PCR Ct values from oropharyngeal or nasopharyngeal swabs on admission were not related to symptom severity.

**Conclusion:**

Ferritin, D-dimer, CRP, NLR, cytokine (IL-18 and IL-10), and cytokine receptor (IL-6, IL1-Ra, and sCD25) test results combined with clinical data can contribute to the early identification of critical COVID-19 patients.

## 1. Background

The first cases of coronavirus disease 2019 (COVID-19) caused by the novel coronavirus 2 (SARS-CoV-2) were reported in China in December 2019 and rapidly escalated to a global pandemic with millions of confirmed cases worldwide, with numbers still rising [1–3]. Most COVID-19 cases are asymptomatic or result in only mild disease although a substantial percentage of patients develop respiratory illnesses requiring hospital care. Pulmonary disease can progress to critical illness with extensive lung damage and hypoxemic respiratory failure requiring prolonged ventilatory support [1]. Therefore, strategies are needed to predict the risk of disease progression. While clinical characteristics and laboratory markers that may be evaluated and used in the emergency setting are under investigation [4, 5] and several prediction scoring models have been developed [6], the identification of patients at high risk of critical illness on admission is still a challenging endeavor for clinicians but is crucial for the screening of patients who might benefit from a more intensive treatment to improve their prognosis. Several COVID-19 studies have shown that increased levels of serum inflammatory markers and proinflammatory cytokines are associated with severe disease [7, 8]. A number of cytokines and chemokines have been implicated in the induction of the “cytokine storm” [9–11], and some, notably interleukin IL 6, are now considered as both prognostic factors and therapeutic targets [12, 13]. Researchers have also suggested that the determination of viral load based on semiquantitative real-time reverse transcription polymerase chain reaction (RT-PCR) cycle threshold (Ct) values from nasopharyngeal swabs may be of prognostic value as lower Ct values have been associated with worse outcomes [14]. Moreover, SARS-CoV-2 viremia has been reported in severe cases [15].

The purpose of our study was to identify routine laboratory, immunological, and virological biomarkers, which, in combination with clinical information, may improve early identification of patients at risk of developing critical illness.

## 2. Methods

### 2.1. Participants and Study Design

Positive nasopharyngeal swab RT-PCR test patients admitted to Son Espases and Son Llàtzer hospitals in Palma de Mallorca (Spain) between 17 April and 20 July 2020 who agreed to participate in the study were included. Blood samples to measure the neutrophil/lymphocyte ratio (NLR) and levels of ferritin, C-reactive protein (CRP), D-dimer, complement components (C3 and C4), lymphocyte subsets, and cytokines, as well as to perform SARS-CoV-2-RT-PCR testing, were obtained within the first 48 hours of admission.

The severity of signs and symptoms developed during hospitalization was categorized as mild (grade 1), severe (grade 2), or critical (grade 3). Mild disease was established when patients had symptoms without pneumonia or with mild pneumonia; severe disease was established when dyspnea was associated with a ≥30/min respiratory rate or <93% blood oxygen saturation or <300 partial pressure of arterial oxygen to fraction of inspired oxygen ratio and/or >50% lung infiltrates within 24 to 48 hours from admission; critical disease was established for cases with respiratory failure, septic shock, and/or multiple organ dysfunction or failure [1]. For each patient, the most severe category developed during hospitalization, which, in all cases, occurred within 72 hours of admission, was selected.

### 2.2. Procedures

Routine blood examinations included leukocyte, neutrophil, and lymphocyte counts (cells ∗ 10^3/*µ*L) and percentages. The serum biochemical tests recorded were ferritin (ng/L), determined by chemiluminescence immunoassay in architect i2000 equipment, CRP (mg/dL), and D-dimer (*µ*g/L), quantified by immunoturbidimetry in architect 16.000. The immunological tests recorded were serum complement levels (C3 and C4), lymphocyte subset cell counts (cells ∗ 10^3/*µ*L) and percentages using flow cytometry, and plasma cytokine levels. We used enzyme-linked immunosorbent assay (ELISA, DIAsource ImmunoAssays, SA, Belgium) to measure serum levels of IL-6, chemiluminescence assay (IMMULITE, Siemens, Germany) to determine serum soluble IL-2 receptor alpha (sIL-2r*α* or sCD25), and a human cytokine magnetic bead panel (Merck Millipore, Billerica, MA, USA) to measure levels of other cytokines associated with cytokine storm: IL-1*β*, IL-1 receptor antagonist (IL-1Ra), IL-6, IL-8, IL-17A, IL-18, IL-22, interferon (IFN), tumor necrosis factor alpha (TNF-*α*), and IL-10.

SARS-CoV-2 was determined in nasopharyngeal swab specimens (within 16 hours of collection) and in plasma samples stored at −70°C until testing. Nucleic acids were extracted using a Hamilton automated extraction platform, and the amplification process was performed in a Bio-Rad CFX96 real-time PCR detection instrument (Bio-Rad, Hercules, CA) using two commercial RT-PCR kits: Allplex 2019-nCoV (Seegene, Seoul, Korea), which detects the presence of 3 target genes E gene, RdRP gene, and N gene and LightMix® Modular SARS-CoV (COVID-19) E-gene (TIB MOLBIOL, Berlin, Germany).

Ct values were recorded for each gene to assess the correlation between semiquantitative viral load values and patterns of disease severity.

### 2.3. Statistical Analysis

Categorical variables were expressed as numbers and percentages, and continuous variables were expressed as mean and standard deviation (SD) or median and interquartile range (IQR) values. Proportions for categorical variables were compared using the *χ*^2^ test. The independent group *t*-test and the Mann–Whitney *U* test were used for the comparison of continuous normally and nonnormally distributed variables, respectively. Normal distribution was studied by plotting histograms and using the Shapiro–Wilk test. A Kruskal–Wallis test was performed to compare the difference between the three groups of patients classified according to disease severity. The receiver operating characteristic (ROC) curve was used to assess the diagnostic value of the biological markers, and the optimal cutoff value providing the best tradeoff between sensitivity and specificity was selected with the Youden index. Univariate and multivariate age-adjusted logistic regression analyses were performed to explore the association between laboratory parameters and the risk of developing critical disease, using the values provided by the Youden index as cutoff points.

All statistical analyses were performed using SPSS (Statistical Package for the Social Sciences) version 22.0 software (SPSS Inc.). Two-sided *p* values of less than 0.05 were considered statistically significant.

## 3. Results

Between 17 April and 20 July 2020, 120 laboratory-confirmed SARS-CoV-2 patients agreed to participate and were included in the study. Fifty-five were women and 65 were men, and median age was 59 years (29–89). COVID-19 was considered mild in 49 patients, severe in 32, and critical in 39 cases. Twenty patients (16.67%) died during hospitalization. The death of 2 of them was not related to COVID-19 but to an associated malignancy. Pulmonary embolism (PE) was diagnosed in 15 patients (12.5%), and of these, 9 had critical COVID-19. Demographic characteristics and comorbidities of SARS-CoV-2-infected patients with mild, severe, and critical disease are presented in Table 1. Compared with patients with mild or severe disease, the number of men among the critically ill was significantly higher (*p*=0.00), they were of older age (*p*=0.036), and concomitant hypertension (*p*=0.036) and diabetes mellitus (*p*=0.023) were more common.

### 3.1. Laboratory Data

**Figure 1:**
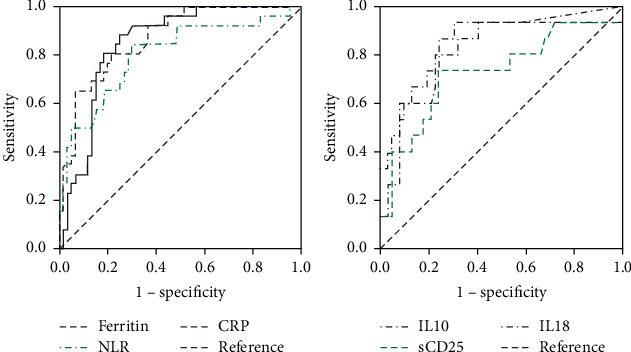
Performances of ROC curves in predicting critical patients for ferritin, CRP, NLR, IL-10, sCD25 (sIL-2r*α*), and IL-18. The univariate and multivariate logistic regression analysis distinguished critical from mild and severe disease patients. Abbreviations: CRP, C-reactive protein; NLR, neutrophil/lymphocyte ratio; and s CD25s (sIL-2r*α*), serum IL2 receptor alpha.

Laboratory markers were tested on admission (Table 2), and their cutoff values were calculated to predict the risk of developing critical COVID-19 (Figure 1). In the univariate analysis, levels of ferritin >370 ng/mL (OR (odds ratio) 16.4, 95% confidence interval (CI) 5.3–50.8), D-dimer >440 ng/mL (OR 5.45, 95% CI 2.36–12.61), CRP >7.65 mg/dL (OR 11.54, 95% CI 4.3–30.8), and NLR >3.77 (OR 13.4, 95% CI 4.3–41.1) were associated with the development of critical COVID-19. In the multivariate analysis, the risk was statistically significant for ferritin (OR 8.1, 95% CI 2.1–30.6), NLR (OR 6.2, 95% CI 1.6–24.0), and CRP (OR 4.9, 95% CI 1.4–17.4), but not for D-dimer.

### 3.2. Immunological Results

Serum complement levels were measured on admission. Median values of C3 and C4 levels in critical COVID-19 patients (group 3) were 116 mg/dL (108.0–127.7) and 26.0 mg/dL (17.0–39.0), respectively, whereas in mild and severe patients (groups 1 and 2), the median values were 125 mg/dL (105.5–145.5) and 26.5 mg/dL (21.0–33.0), respectively (*p* > 0.05). Low C4 levels (<20 mg/dL) were found in 5/15 (33.3%) patients with PE compared with 22/105 (20.9%) patients without PE (*p* > 0.05) and in 10/39 (25.6%) patients with critical disease compared with 9/49 (18.4%) patients with mild disease (*p* > 0.05).

Levels of IL-6, sIL-2r*α* (sCD25), IL-1*β*, IL-1Ra, IL-8, IL-17A, IL-18, IL-22, IFN, IL-10, and TNF-*α* were analyzed (Table 3). IL-6, IL-10, sIL-2r*α*, IL-1Ra, and IL-18 levels were significantly elevated on admission in patients who went on to develop a critical disease. A cutoff value for each of these markers was determined to predict the risk of developing critical COVID-19 (Figure 1). IL-6 >142.5 pg/mL, IL-10 >10.8 pg/mL, IL1*β* > 4.68 pg/mL, sIL-2r*α* >804.5 pg/mL, IL-1Ra >88.4 pg/mL, and IL-18 > 144 pg/mL values were associated with the development of critical COVID-19 in the age-adjusted univariate analysis. This association was confirmed only for IL-10, sIL-2r*α*, and IL-18 in the age-adjusted multivariate analysis (Table 4).

Lymphocyte subsets were analyzed in peripheral blood in a subgroup of patients on admission. The total number of T, B, and natural killer (NK) cells was significantly decreased in patients with more severe disease (Table 5). No univariate or multivariate analyses were performed due to the small number of patients with critical COVID-19.

### 3.3. Microbiological Study

Oropharyngeal or nasopharyngeal swab samples obtained from 89 patients on the day of admission were tested for SARS-CoV-2 by RT-PCR either targeting E, N, and RdRP genes (72 patients) or E gene alone (17 patients). No significant differences in median Ct values according to symptom severity were observed (Table 6). No statistically significant differences between Ct values and number of days from symptom onset on admission or mortality were found either. Ct values > 34 (associated with a low viral load) for the E gene on hospital admission were found in 16/89 (17.9%) patients; 2 had critical COVID-19, and 5 had severe COVID-19. Additionally, the N and RdPR genes were studied in 8/16 of these patients and a Ct value > 34 was found in 3 cases. SARS-CoV-2 RT-PCR was performed in blood samples from 73 patients. All the results were interpreted as negative except for 3 patients who had indeterminate results (only a Ct > 38 for the N gene was detected), and all 3 died.

## 4. Discussion

Our study has identified several biomarkers that may be used in combination with clinical characteristics to better evaluate the severity of COVID-19 and optimize therapeutic management strategies. In addition, we suggest cutoff values for each of these markers for the prediction of the risk of developing critical COVID-19. sCD25, IL-1Ra, and IL-18 are of special interest as they have rarely been described as prognostic factors in COVID-19.

CRP, D-dimer, and ferritin have been described as hyperinflammatory state and disease severity markers early in the pandemic [7, 16, 17], and COVID-19 guidelines have suggested that measuring their levels may have prognostic value [18]. Ferritin may be produced by activated pulmonary macrophages, and COVID-19 systemic inflammation is now considered as part of the spectrum of hyperferritinemic syndromes as significant increases in ferritin levels have been found in severe compared with nonsevere COVID-19 patients [19–22]. Similarly, CRP and D-dimer have been identified as prognostic markers, with suggested cutoff values published in several studies: CRP levels >4.96 mg/dL and D-dimer levels >2600 ng/mL have been associated with critical illness in a study in China [7], whereas D-dimer levels >500 ng/mL or >1000 ng/mL have been identified as significant risk factors for death in other studies [17, 23, 24]. However, in our study, the cutoff value of 440 ng/mL for D-dimer was associated with a higher risk of developing critical disease only in the univariate analysis and not in the multivariate analysis. Moreover, it was not associated with PE, since D-dimer concentrations >440 ng/mL on admission were only observed in 9/15 patients who later developed PE during hospitalization. NLR has already proven its prognostic value in cardiovascular and inflammatory diseases, several types of cancer, and certain bacterial diseases [25]. Since the beginning of the COVID-19 pandemic, lymphopenia and increased NLR have been widely described as severity markers [9, 16, 17]. Different mechanisms have been proposed for COVID-19-related lymphopenia: T-cell exhaustion, apoptosis, pyroptosis, and a direct virus cytopathic effect [26]. Flow cytometry evaluation was useful in our study to investigate lymphopenia. In line with results from other studies, we found a significant reduction of all lymphocyte subsets, CD3+, CD4+, and CD8+ T cells (with no inversion in the CD4+/CD8+ ratio), NK cells, and B lymphocytes, in severe/critical compared with mild patients [16, 27, 28]. In addition, CD3+CD8+ T cell counts ≤75 cells/*μ*L have been associated with death [23].

Other immunological markers were also investigated. The activity of the complement system was assessed since hypocomplementemia has been reported in several viral infections and activation of the complement system as well as vascular deposition of complement components have been found in SARS-CoV thrombotic microangiopathy. Neither our study nor a previously published report found lower C3 or C4 levels in severe COVID-19 patients [29], although a relationship between PE and low C4 levels cannot be excluded. However, a recent study assessed the role of the complement system and found higher levels of C3a, C3c, and terminal complement complex in Intensive Care Unit (ICU) COVID-19 patients compared with non-ICU COVID-19 patients [30].

Eleven cytokines or their receptors were tested on admission in the 120 patients, and IL-6, IL-10, IL1*β*, IL-1Ra, sIL-2r*α* (sCD25), and IL-18 were found to be of prognostic value.

High serum levels of IL-6 have been widely described as a hallmark of severity in COVID-19 [9, 11, 16, 31, 32]. IL-6 plays a central role in the cytokine storm as it induces cytotoxic T lymphocytes, promotes Th17 cell lineage differentiation, inhibits regulatory T cells, and activates B cells and antibody production. A recent systematic review and meta-analysis [33] concluded that threefold higher serum IL-6 levels were found in patients with severe COVID-19 compared with those with noncomplicated disease. Increased levels were significantly associated with adverse clinical outcomes, including ICU admission, acute respiratory distress syndrome, and death. Furthermore, several studies have suggested IL-6 and IL-6 receptor antagonists may be of benefit in the management of critical COVID-19 patients [12, 21, 34].

We found that both IL-1Ra and IL-10 anti-inflammatory cytokines were significantly elevated in critical compared with moderate or severe cases. IL-10 has been described early in the pandemic as a marker of severe disease [9, 31]. IL-1Ra produced by activated macrophages is a competitive antagonist for IL-1 and controls inflammatory responses, modulating the production of other inflammatory cytokines. IL-1Ra has been previously investigated in several small studies and increased levels have been found in severe clinical cases [35, 36], supporting its role as an important marker of disease severity.

IL-18 is a proinflammatory cytokine that facilitates IFN-*γ* production by Th1 cells in conjunction with IL-12 and activation of CD8+ T cells. In our study, significantly higher IL-18 levels were found in critical patients compared with moderately or mildly ill patients. A correlation between IL-18 and COVID-19 severity had only been found in a small study [8], but a more recent study suggested this cytokine might be of prognostic value [37].

Serum sIL-2r*α* (sCD25) is considered an important disease marker in hemophagocytic syndromes/hemophagocytic lymphohistiocytosis [38]. However, its value as a prognostic factor in COVID-19 has been rarely reported. A small preliminary study in China with 21 patients found significantly increased serum sCD25 levels in critical patients compared with patients with moderate disease [16], and the authors suggested that sCD25 may act as a T-cell negative regulatory factor contributing to lymphopenia. Other investigators have suggested the relevance of the sIL-2r*α*/lymphocyte index for early identification of severe COVID-19 and prediction of the clinical progression of the disease [39]. In our study, sCD25 levels were accurate in estimating the risk of complicated disease, as a >804.5 pg/mL cutoff value yielded 73.3% sensitivity and 76.0% specificity for developing critical COVID-19.

Finally, virological markers were studied as prognostic factors for the development of severe disease, but our results failed to confirm any association. We did not find a correlation between RT-PCR Ct values from nasopharyngeal swab specimens and severity of the disease. Previous studies have reported conflicting results: a correlation between lower Ct values (representing higher viral loads) in respiratory samples and greater disease severity has been shown in certain studies [40–43], while other non-SARS-CoV-2 respiratory virus studies have not shown such correlation [14, 40–42, 44]. SARS-CoV-2 blood testing was negative in all cases, except for 3 patients who had indeterminate results and all 3 died. The detection of SARS-CoV in plasma was associated with critical disease in the 2003 SARS pandemic [45], but COVID-19 studies have been scarce. One study detected SARS-CoV-2 viremia in up to 41% of patients admitted in a hospital in Zhejiang Province, China [46].

Our study has some important limitations: it took place in a very specific geographic area (two hospitals in the Mediterranean island of Majorca), and the number of patients was small. In addition, patients were admitted at different times during the COVID-19 pandemic and received different therapies, which may have altered the outcomes.

In conclusion, ferritin, D-dimer, CRP, NLR, cytokines (IL-6, IL-18, and IL-10), and cytokine receptors (IL-1Ra and sIL-2r*α* [sCD25]) in combination with clinical information may aid the early identification of patients at risk of critical COVID-19. These patients would be eligible for a more intensive therapy. Further studies with larger cohorts are warranted to confirm the ability of these biomarkers in predicting critical COVID-19.

## Figures and Tables

**Table 1:** Demographic characteristics and comorbidities of SARs-CoV-2-infected patients with mild, severe, and critical disease.

	Mild (*n* = 49)	Severe (*n* = 32)	Critical (*n* = 39)
Age, years, median (IQR)	51 (44.5–64.5)	61 (48.5–75.5)	66 (56–73)
Sex, women/men	32/17	16/16	7/32
Days from symptom onset on admission, median (IQR)	10 (4–16)	7 (5–13.7)	7 (5–10)
Chronic cardiovascular disease	9	4	8
Hypertension	13	7	17
COPD	6	3	5
Renal disease (^*∗*^)	1	2	1
Diabetes	5	3	10
Obesity (body mass index >30)	11	2	10
Pregnancy	2	0	1
Liver cirrhosis	1	1	2
Neurological disease (^†^)	7	2	5
Active solid/hematological malignancy	4	0	1
HIV infection	3	0	1
Active smoker	12	3	7

Abbreviations: COPD, chronic obstructive pulmonary disease; HIV, human immunodeficiency virus; SARS-CoV-2, severe acute respiratory syndrome coronavirus 2. (^*∗*^) included advanced-stage renal disease, transplant recipient, or hemodialysis patients. (^†^) included cerebral vascular disease and Parkinson disease.

**Table 2:** Comparison of laboratory markers in mild, severe, and critical COVID-19, median and IQR.

	Mild (*n* = 47)	Severe (*n* = 32)	Critical (*n* = 38)	*p* value
Ferritin (ng/mL)	117 (69.0–300.0)	437.0 (215.0–709.0)	1107 (582–2435)	*p* < 0.001
D-dimer (ng/mL)	218 (106.7–354.2)	291.0 (187.5–2008.0)	533.0 (290.0–2153.0)	*p* = 0.007
NLR	2.5 (1.9–3.5)	3.6 (2.5–6.8)	8.1 (4.1–13.7)	*p* < 0.001
CRP (mg/dL)	0.9 (0.3–4.7)	5,6 (2.8–11.8)	17.4 (10.0–23.7)	*p* < 0.001

Abbreviations: COVID-19, coronavirus disease 2019; CRP, C-reactive protein; IQR, interquartile range; NLR, neutrophil/lymphocyte ratio.

**Table 3:** Comparison of cytokine levels on admission in mild, severe, and critical COVID-19 (median and IQR).

	Mild, *n* = 41	Severe, *n* = 31	Critical, *n* = 38	*p* value
**IL-6**	28.0 (13.0–76.0)	36.0 (18.0–87.0)	218.5 (47.6–819.7)	*p* **<** **0.001**
**IL-10**	0.0 (0.0–14.2)	4.1 (0.0–36.5)	63.1 (21.5–112.9)	*p* **<** **0.001**
**sIL-2r*α*** (sCD25)	519 (364.2–768.0)	701 (474.0–795.0)	972 (579.0–1338.0)	*p* **=** **.012**
IFN	5.1 (1.4–13.0)	11.7 (1.6–39.7)	13.9 (3.1–33.7)	n.s.
**IL1*β***	2.9 (0.9–2.0)	7.9 (1.3–12.3)	11.2 (5.1–18.5)	*p* **=** **0.001**
**IL-1Ra**	47.7 (24.6–96.5)	54.8 (32.0–125.4)	104.5 (49.3–333.0)	*p* = 0.001
IL-8	42.8 (14.5–93.9)	51.5 (20.5–116.2)	49.7 (21.7–109.4)	n.s.
IL-17A	0.0 (0.0–2.7)	0.0 (0.0–3.8)	0.0 (0.0–9.17)	n.s.
**IL-18**	51.0 (17.9–92.4)	46.8 (32.8–93.9)	181.8 (87.3–307.5)	*p* **<** **0.001**
IL-22	(0.0–0.0)	0.0 (0.0–72.1)	0.0 (0.0–0.0)	n.s.
TNF	31.8 (19.4–54.9)	37.7 (22.7–63.3)	50 .7 (35.7–79.3)	n.s.

Abbreviations: COVID-19, coronavirus disease 2019; IFN, interferon; IL-1Ra, IL-1 receptor antagonist; n.s., not significant; TNF, tumor necrosis factor; sIL-2r*α*, serum IL-2 receptor alpha (sCD25).

**Table 4:** IL-6, IL-10, sIL-2r*α* (sCD25),IL1*β*, IL-1Ra, and IL-18 cutoff values and ORs (95% CI) for the risk of developing critical COVID-19 in the age-adjusted univariate and multivariate analyses.

Variable	Cutoff (pg/mL)	OR (95% CI) univariate model	OR (95% CI) multivariate model
IL-6	142.5	8.76 (3.56–21.54)	—
IL-10	10.8	20.73 (5.83–73.71)	8.09 (1.73–37.97)
sIL-2r*α* (sCD25)	804.5	12.46 (4.00–38.8)	11.07 (2.39–51.32)
IL1*β*	4.68	4.57 (1.81–11.53)	—
IL-1Ra	88.4	4.14 (1.82–9.41)	—
IL-18	144.0	16.10 (5.83–44.46)	10.68 (2.87–39.69)

Abbreviations: CI, confidence interval; COVID-19, coronavirus disease 2019; IL-1Ra, IL-1 receptor antagonist; OR, odds ratio; sIL-2r*α*, serum IL-2 receptor alpha (sCD25).

**Table 5:** Lymphocytes CD3, CD4, CD8, CD4/CD8 ratio, CD19, and NK cells in patients with mild, severe, and critical COVID-19 (median and IQR).

	Mild (*n* = 39)	Severe (*n* = 23)	Critical (*n* = 14)	*p* value
CD3	1411 (986–2137)	938 (639–1426)	507 (238–938)	*p* < 0.05
CD4	950 (653–1390)	794 (463–1034)	256. (163–594)	*p* = 0.00
CD8	478 (317–685)	327 (132–408)	190 (63–253)	*p* = 0.00
CD19	256 (170–358)	166 (84–218)	93 (74–158)	*p* < 0.05
NK	170 (130–287)	143 (72–320)	65 (37–123)	*p* < 0.05
CD4/CD8	2.2 (1.5–2.9)	2.1 (1.5–3.4)	2.2 (1.0–2.8)	*p* > 0.05

Abbreviations: COVID-19, coronavirus disease 2019; NK, natural killer.

**Table 6:** E, N, and RdRp median Ct values and IQRs according to symptom severity.

	E gene (*n* = 89)	N gene (*n* = 72)	RdRP gene (*n* = 72)
Mild	29.7 (22.2–35.3)	31.9 (25.7–36.2)	25.1 (20.5–32.5)
Severe	27.6 (19.4–33.1)	28.0 (21.9–33.9)	27.0 (20.9–34.1)
Critical	26.4 (21.9–29.5)	29.9 (25.5–31.2)	26.9 (22.9–30.4)

Abbreviations: Ct, cycle threshold; IQR, interquartile range.

## Data Availability

The datasets used and analyzed during the current study are available from the corresponding author on request.

## Abbreviations

Ct values: Cycle threshold values
CRP: C-reactive protein
IL-1Ra: IL-1 receptor antagonist
IFN: Interferon
NK: Natural killer cells
NLR: Neutrophil/lymphocyte ratio
PE: Pulmonary embolism
sIL-2r*α* or sCD25: Soluble IL-2 receptor alpha
TNF-*α*: Tumor necrosis factor alpha.
